# *Streptococcus mutans* Growth and Resultant Material Surface Roughness on Modified Glass Ionomers

**DOI:** 10.3389/froh.2020.613384

**Published:** 2020-11-17

**Authors:** Riaan Mulder, Ernest Maboza, Rukshana Ahmed

**Affiliations:** ^1^Restorative Dentistry, The University of the Western Cape, Cape Town, South Africa; ^2^Dental Research Laboratory, The University of the Western Cape, Cape Town, South Africa

**Keywords:** antimicrobial effect, chitosan, glass ionomer, nanodiamond, *Streptococcus mutans*, surface roughness

## Abstract

The present study investigate the optical density of *Streptococcus mutans* (*S. mutans*) at 450 nm (OD450 nm) as well as the change in surface roughness of three commercially available chitosan- and nanodiamond-modified glass ionomers. The results indicated that the optical density of *S. mutans* OD450 nm decreased significantly (*p* < 0.0001) from 0 h through 2–4 h for each of the control materials. The lowest *S. mutans* OD450 nm was noted for Fuji IX followed by Ketac Universal. Riva Self Cure had the largest increase in the *S. mutans* OD450 nm. The control materials and their chitosan/nanodiamond modifications showed significant growth at 6 h compare to the preceding time periods of 2 and 4 h. The materials Fuji IX, Fuji IX modified with 5% Nanodiamonds, Fuji IX modified with 10% Chitosan and Ketac Universal modified with 10% Chitosan performed the best with regard to the bacterial reduction. Only the chitosan modifications showed an increase in the surface roughness after 24 h of exposure to the *S. mutans*. The chitosan and the nanodiamond modifications provided the best disruption of the *S. mutans* biofilm formation.

## Introduction

*Streptococcus mutans* (*S. mutans*) has been identified as one of the main role players in the development of dental caries [1]. *S. mutans* form part of the phylogenetic tree of 34 species of Streptococcus species that have been classified into five phylogenic groups per viridians [2]. *S. mutans* has been presented to be one of the most virulent bacterial species in the caries process [3, 4] and has been implicated in the progression of dental caries [1, 5, 6]. The metabolism of carbohydrates [7] by *S. mutans* results in its adherence to tooth structure and subsequent high aciduricity [8] and acidogenecity [6, 9, 10]. Plaque samples have been shown to contain high levels of l-lactic isomer that is the main acid produced by *S. mutans* [11]. Glass ionomer cements (GICs) has become an option as a restorative material for high caries risk patients, since they are known to have an inhibitory influence on *S. mutans* [12] and have been shown to reduce their acid production [13, 14].

Various antibacterial materials have been incorporated into GICs in order to increase their antibacterial activity. These included but not limited to benzalkonium chloride [15], cetrimide [16], cetylpyridinium chloride [16], chlorhexidene [17], zinc sulfate [18], and silver or zinc zeolites [19].

Ching et al. [20], completed a literature review of 22 studies on various modifications of GICs that enhance their antibacterial activity. Many of the studies reviewed did not demonstrate a significant improvement in the antibacterial properties [20]. The studies where the chitosan [21–23] was incorporated into the liquid component of the GIC in a 5 and 10% volume per volume percent (v/v %) in relation to the control material showed favorable results [22, 23].

There are however various factors that influence the interaction of the micro-organisms with tooth structure and the restorative materials. The bacterial adherence, colonization, and growth of bacteria on the restoration and tooth structure have been linked to the surface roughness. Bacterial colonization on surfaces with a surface roughness (Ra) of more than 0.2 μm has been detected to be significant [24]. High levels of *S. mutans* were detected in plaque from saliva and the surfaces of carious teeth as well as sound teeth [1]. The interaction of *S. mutans* with dental materials has been of importance for dental research. Although GICs have been identified to have antibacterial properties, a bacterial biofilm can form on the surface of GICs [25]. The modification of GICs with chitosan- and nanodiamond particles in the powder of the GICs has never been assessed. This study therefore investigated the changes in the *S. mutans* OD450 nm as well as the resultant surface roughness of the chitosan- and nanodiamond-modified GICs.

The aims of the study were to investigate the change in surface roughness before and after 24-h exposure, as well as the change in the OD450 nm of the *S. mutans* at different time intervals. The hypothesis was that chitosan- and nanodiamond-modified GICs would reduce the *S. mutans* OD450 nm with no effect on the surface characteristics of the modified GICs.

## Materials and Methods

### Material Modification

Three commercially available GICs namely FN: Fuji IX GP hand-mix (GC Corp, Tokyo, Japan; Batch: 1503231); KU: Ketac Universal hand-mix; (3M ESPE, Seefeld, Germany; Batch: 583514) and RSC: Riva Self Cure hand-mix (SDI Limited, Australia; Batch: 62657V) were used in this study. These GICs were modified in the powder phase with either 5 or 10% chitosan or nanodiamond particles.

The nanodiamond particles were commercially available and produced by detonation (PlasmaChem, Germany; item: PL-D-G01). The nanodiamonds are produced by detonating 60% weight percentage TNT (C_6_H_2_(NO_2_)_3_CH_3_) and a 40% weight percentage of hexogen (C_3_H_6_N_6_O_6_) in a negative oxygen environment. The detonation occurs inside a metallic chamber with an atmosphere of N_2_, CO_2_ and liquid or solid water [26]. The average particle size is 4–6 nm and the particles generally present in non-fused diamond clusters. The carbon purity of >98% has an enhanced stability (5 w/v%) in a water suspension and carries a −55 ±5 mV Zeta potential.

The three GICs were modified in the powder phase per weight percentage (w/w%) by adding 5% or 10% of a commercially available chitosan particles (Merck, item 448877) or nanodiamond particles. The GICs modified with chitosan- and nanodiamond particles were produced by placing the GICs powder in an airtight high density polyethylene (HDPE) container. The containers were subsequently clamped in a beaker shaker for 2 h to ensure complete mixing of the two powders.

After modification with the chitosan- and nanodiamond particles, samples from the fifteen GICs were prepared in accordance with the manufacturers' powder/liquid ratios: (1) FN GIC powder [FN]; (2) FN GIC powder modified with 5-wt% chitosan particles [FN5%CH]; (3) FN10%CH; (4) FN5%ND; (5) FN10%ND; (6) KU; (7) KU5%CH; (8) KU10%CH; (9) KU5%ND; (10) KU10%ND; (11) RSC; (12) RSC5%CH; (13) RSC10%CH; (14) RSC5%ND; (15) RSC10%ND.

### Sample Preparation

The powder/liquid ratio prescribed by the manufacturer was followed and confirmed on a desktop analytical balance (Metler AE240 analytical balance, Columbus, OH, USA) by first dispensing the powder followed by the liquid. This ensured that the manufacturer's recommended powder/liquid ratio was maintained for all the GICs used in this study. Disc-shaped specimens of GIC were prepared (7 ± 0.1 mm diameter and 2 ± 0.1 mm thickness) by packing the GICs into a Teflon split mold and covering it on both sides with polyester strips followed by a glass microscope slide and leaving it to set at room temperature (± 23°C) for 1 h. After setting, the polyester strips and microscope slides were removed and the specimens were stored for 24 h at 37°C and 100% humidity in a temperature controlled chamber. Samples were treated with wet silicon-carbide paper on both sides (1,200 grit sand paper as ISO9917-1:2007, as per the acid erosion test). Carlén et al. [27], illustrated that after polishing a GIC restoration, the protein/bacterial binding and the surface roughness did not change in relation to the GIC prior to polishing. Despite their result [27] this study design still retained a standard surface roughness (Ra) and it was ensured during the surface preparation through the assessment of the surface roughness for each group of GICs to be within Ra ± 2 μm.

### Surface Roughness

Two surface roughness (Ra) measurements were recorded namely, before exposure to *S. mutans* [known as “Ra 0 (zero) h] and after exposure to *S. mutans* (known as Ra 24 h). The operator was blinded to the GICs modification and material that was assessed. An independent researcher responsible for the blinding of the material samples assessed that all the samples per group complied with the Ra ± 2 μm. The Ra of all the materials were measured with a Leeb surface roughness tester with a standard sensor (Model Leeb432, Chongqing Leeb Instrument co., Ltd.). The Ra tester has a wide measuring range between 0.005 and 16 μm for surface roughness.

The testing parameters in this study were: surface roughness (Ra), the filter option was adjusted to the Gauss setting, the assessment length (λc) at 0.8 mm × *n* 5 (assessment length (Ln = lr × *n*); Ln = 3.2 mm). The standard stylus has a natural diamond at a 90° cone angle with a 5 μm tip radius. The stylus applied a force to the sample of <4 mN. The traveling speed (Vt) for the above parameters was 0.135 mm/s and the measurement accuracy was ± 10%.

Two parallel lines were recorded 2 mm apart with two lines perpendicular to that (*n* = 4) per side of each sample. The aforementioned measurements for both sides of the sample (*n* = 8) were therefore recorded and the average of the eight measurements was used as the mean Ra per specimen for time period Ra 0 h. The GICs samples were re-assessed after 24 h subsequent to the 24-h exposure to the tryptic soy broth containing *S. mutans* (TSB/*S. mutans* broth) at Ra 24 h. Subsequent to removing the GICs samples from the TSB/*S. mutans* growth medium, a 10-s vortex in 5 mL de-ionized water was completed to remove any *S. mutans* biofilm and bacteria from the surface of the GICs. The aforementioned samples (*n* = 8) for Ra readings were then repeated. The mean percentage difference was calculated for the five samples per GICs group in relation to the Ra 0 h value. A positive percentage value indicated an increase in Ra from Ra 0 h to Ra 24 h.

### Sterilization

Sterilization of the GICs samples were completed after the first surface roughness readings (Ra 0 h) was completed. The GICs specimens and the 5 mL cryotubes were sterilized with ethylene oxide gas (Steri-Vac 4XL gas sterilizer, Model 400DGP, 3M, St Paul, MN, USA). The ethylene oxide gas was used, since the rise in temperature to 120°C in the steam autoclave could alter the surface due to moisture absorption and the high temperature, causing surface cracks in the GICs that was not part of normal maturation. The development of cracks would alter the surface roughness.

### Reconstitution of *S. mutans*

*S. mutans* bacteria (ATCC 25175) was reconstituted in brain heart infusion (BHI broth) for 24 h at 37°C and streaked on a contact plate (TSA LTHth-ICR, Merck Life Science GmbH; Eppelheim; Germany; Batch 140477) in order to isolate a single *S. mutans* colony.

The single *S. mutans* colony (ATCC 25175) taken from the agar plate was grown overnight at 37°C under 5% CO_2_ conditions in a BHI broth. The overnight culture was added in 5 μL at a time to the sterile phosphate buffered saline (PBS). The McFarland 0.5 was established and assessed with the DensiCHECK plus measuring device at a 580 nm wavelength (BioMerieux Inc, Hazelwood, Missouri, USA). The McFarland 0.5 was equivalent to a concentration of 1.5 × 10^8^ *S. mutans* cells/mL in the PBS solution. One thousand milliliters of tryptic soy broth (TSB) was manufactured using 30 g of TSB (TSB CM0129; Oxoid Ltd.; Basingstoke; Hampshire; UK) mixed with 1,000 mL of de-ionized water and sterilized in an autoclave at 121°C for 15 min. After the broth had cooled to 23°C, 50 μL of the McFarland standard 0.5 *S. mutans*/PBS suspension (1.5 × 10^8^ *S. mutans* cells/mL in PBS) was added to 5 mL of TSB in the sterile cryotubes (TSB/*S. mutans*). The inoculated 5 mL TSB/S. *mutans* and the five GIC samples per material were placed in an orbital shaking incubator (MRC, Holon, Israel) at 37°C with a speed of 120 rotations per hour. The *S. mutans* control was sterile cryotubes inoculated with the same *S. mutans*, but no GICs were added to these cryotubes. The *S. mutans* control would provide the growth pattern in order to evaluate the effect of the various GICs investigated in this study.

### Colorimetric Assay (XTT Based) of Cell Proliferation and Viability

The changes in the *S. mutans* optical density measured at 450 nm (OD450 nm) of *S. mutans* were evaluated at 0, 2, 4, 6, and 24 h. The 5 mL cryotubes was tipped twice at 180° and 300 μL from the 5 mL cryotubes TSB/*S. mutans* bottle was equally divided between three wells on a 96-well plate at 37°C [28]. For the XTT analysis, each well of the 96-well plate contained 100 μL TSB/*S. mutans* and 50 μL of a XTT Cell Proliferation kit II (Roche Diagnostics GmbH, Mannheim, Germany). The XTT labeling reagent and coupling agent were mixed in accordance with the manufacturers' instructions (Roche Diagnostics GmbH, Mannheim, Germany). The spectrophotometer measurements were taken after 4 h of incubation at 450 nm (Smart Microplate Reader, Model SMR16.1. USCN life science kit Inc, Wahan, China). At optical density of 450 nm, the mitochondrial activity was determined with the aid of formazan dye.

### Statistical Analysis

The statistical analysis for the *S. mutans* bacterial OD450 nm was completed in two stages. Stage 1: The change in the bacterial OD450 nm for 2, 4, 6, and 24 h was assessed against the 0 h *S. mutans* OD450 nm within the three materials (FN, KU and RSC) and then with their respective chitosan and nanodiamond modifications. The one way ANOVA with linear mixed modeling was completed. The data were considered to be longitudinal for which the linear mixed effects models have been developed (*Modern Applied Statistics with S*, by W.N. Venables and B.D. Ripley).

Stage 2: One-way ANOVA was used with the *p*-value corresponding to the F-statistic of one-way ANOVA *p* < 0.05. This suggests that one or more treatments for *S. mutans* OD450 nm were significantly different between the control and the three materials (FN, KU, and RSC) and their chitosan and nanodiamond modifications. The *post-hoc* Holm-Bonferroni formula for the first-ranked *p*-value was used to determine the true significance for 0, 2, 4, 6, and 24 h in relation to the control *S. mutans* OD450 nm for the same time periods.

The change in surface roughness between 0 and 24 h were calculated as a percentage by using the following formula: [(100/Ra 0 h) × (Ra 24 h)] – [100].

## Results

### Surface Roughness Change (Ra)

**Figure 1:**
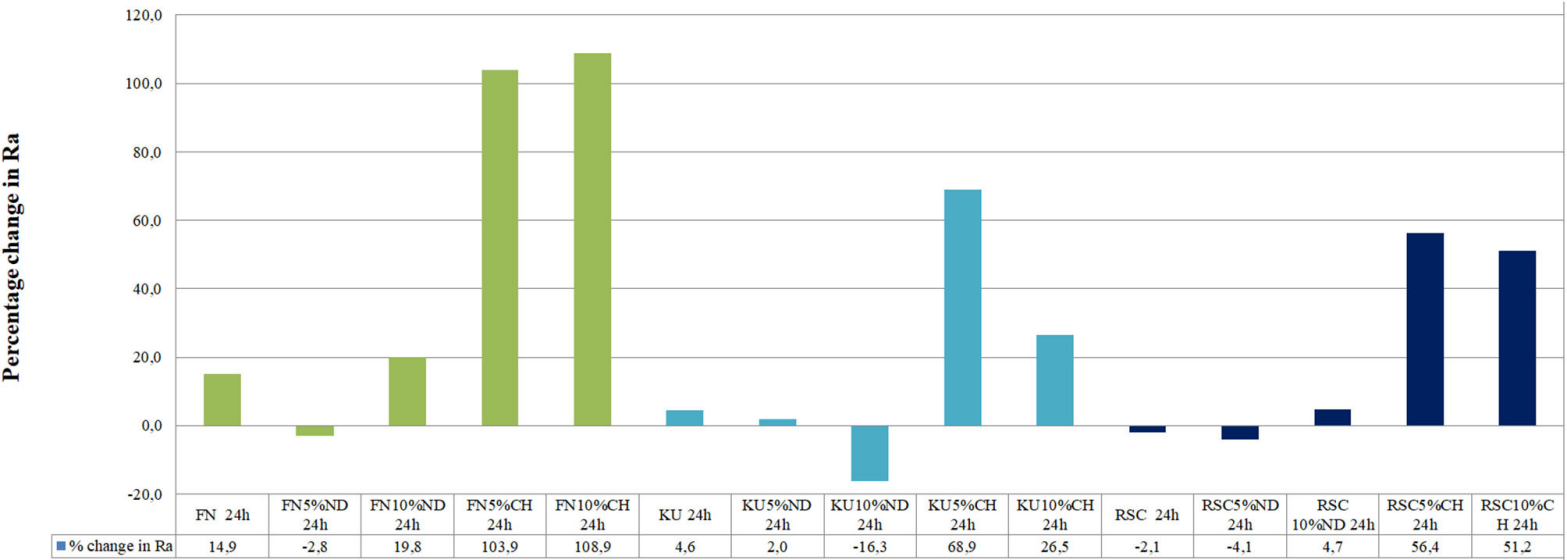
Percentage of surface roughness change of the GICs surfaces from 0 to 24 h.

Figure 1 illustrates the difference of the surface roughness between the start of the experiment (0 h) and the end of the study (24 h). A positive percentage change of the surface roughness from 0 to 24 h represents an increase in surface roughness. Table 1 indicates the precise surface roughness values as recorded for each material at 0 and 24 h with significant differences presented by a *p* < 0.05.

**Table 1:** Change in surface roughness values (μm) from 0 to 24 h for each material.

	**FN**	**FN5%ND**	**FN10%ND**	**FN5%CH**	**FN10%CH**
Ra 0 h	0.971	1.371	0.990	1.023	1.650
Ra 24 h	1.116	1.333	1.187	2.085^∧^	3.447^∧^
	**KU**	**KU5%ND**	**KU10%ND**	**KU5%CH**	**KU10%CH**
Ra 0 h	0.833	0.736	0.924	1.196	1.581
Ra 24 h	0.871	0.751	0.774	2.021^∧^	2^∧^
	**RSC**	**RSC5%ND**	**RSC10%ND**	**RSC5%CH**	**RSC10%CH**
Ra 0 h	1.366	1.163	1.244	1.617	1.797
Ra 24 h	1.338	1.115	1.301	2.528^∧^	2.716^∧^

∧ *indicates significant change p < 0.05 within the respective material group*.

Comparison of the optical density (OD450 nm) absorbance values from the XTT test is indicated in Table 2. The significant differences between the material and the *S. mutans* control in Table 2 are indicated per time period. The ANOVA of the OD450 nm achieved in the XTT test of *S. mutans* identified significant differences between the three control materials (FN; KU; RSC: *p* < 0.0001). This difference indicates significant differences between the amounts of viable streptococci at 24 h for the three control materials. The lowest *S. mutans* OD450 nm was noted for FN (0.783) followed by KU (0.927). RSC (1.056) had the largest *S. mutans* OD450 nm.

**Table 2:** Comparison of the mean OD450 nm of *S. mutans* control and the dental materials per time period.

**Material**	**Hours**				
	**0**	**2**	**4**	**6**	**24**
*S. mutans* control	0.717	0.607	0.601	0.680	0.944
FN	0.726	0.563	0.571	0.658	0.783^*^
FN5%CH	0.593^*^	0.555	0.542	0.653	0.762^*^
FN10%CH	0.635	0.553	0.552	0.625	0.689^*^
FN5%ND	0.623	0.567	0.528	0.629	0.759^*^
FN10%ND	0.686^∧^	0.711	0.670	0.753	0.831^*^
KU	0.620^*^	0.570	0.588	0.608	0.927
KU 5%CH	0.616^*^	0.548	0.569	0.617	0.903
KU 10%CH	0.597^*^	0.531^∧^	0.611	0.678	0.842^*^
KU 5%ND	0.635^*^	0.566	0.565	0.577^*^	0.930
KU 10%ND	0.570^∧^	0.539	0.536	0.650	0.906
RSC	0.587^*^	0.536	0.515^∧^	0.618	1.056
RSC 5%CH	0.586^*^	0.535	0.552	0.630	1.045
RSC 10%CH	0.558^*^	0.525	0.547	0.633	1.021
RSC 5%ND	0.533^*^	0.543	0.557	0.598^∧^	0.973
RSC 10%ND	0.668	0.592	0.516^∧^	0.635	1.081

* *indicates significant difference (p < 0.01) with the S. mutans control for the same time period*.

∧ *indicates significant difference (p < 0.05) with the S. mutans control for the same time period*.

### *S. mutans* and the Control Materials

Table 2 illustrates the *S. mutans* growth incubated with the GIC material over time. The *S. mutans* OD450 nm decreased significantly (*p* < 0.0001) from0 h through 2–4 h for each material. At 6 h, the *S. mutans* OD450 nm increased when compared to 0, 2, and 4 h. There were no significant differences in the OD450 nm at any time period (0, 2, 4, and 6 h) between the three control GIC materials with *p* = 0.9969. At 24 h there were significant differences between all three control materials (*p* < 0.0001). The lowest *S. mutans* OD450 nm was noted for FN followed by KU. RSC had the largest *S. mutans* OD450 nm.

### Nanodiamond Modification of GICs

The ANOVA of the OD450 nm achieved in the XTT test of the control material compared to its respective nanodiamond modification identified no significant differences (*p* > 0.05). There were no significant differences in the *S. mutans* OD450 nm at any time period (0, 2, 4, 6, and 24 h) between the various comparison pairs: FN vs. FN5%ND; FN vs. FN10%ND; KU vs. KU5%ND; KU vs. KU10%ND; RSC vs. RSC5%ND; RSC vs. RSC 10%ND.

### Chitosan Modification of GICs

The ANOVA of the OD450 nm achieved in the XTT test of the control material compared to its respective chitosan modification identified no significant differences (*p* > 0.05). There were no significant differences in the *S. mutans* OD450 nm at any time period (0, 2, 4, 6, and 24 h) between the various comparison pairs: FN vs. FN5%CH; FN vs. FN10%CH; KU vs. KU5%CH; KU vs. KU10%CH; RSC vs. RSC5%CH; RSC vs. RSC10%CH.

### Nanodiamond vs. Chitosan Modification of GICs

The ANOVA of the OD450 nm achieved in the XTT test of the GIC modified with nanodiamond particles vs. chitosan, identified no significant differences (*p* > 0.05).

## Discussion

The hypothesis was not accepted that chitosan-modified GICs would reduce the *S. mutans* OD450 nm at 24-h for KU and RSC with no significant effect on the surface characteristics of these two chitosan- and nanodiamond modified GICs. It was however accepted for FN GICs modified with nanodiamonds.

Nanodiamonds combined with GICs posed the potential for enhancing the antibacterial activities of GICs. Table 2 illustrates that all the assessed commercial GICs (FN, KU, and RSC) showed a reduction in the *S. mutans* OD450 nm in relation to the control bacteria. Although it was not significantly reduced for FN when compared to the control for 2, 4, and 6 h, there was a significant reduction at the 24-h growth period. The growth period at 24-h is important, since this plaque biofilm will cause an increased risk for caries. This was the general trend for the chitosan and nanodiamond modifications as well. KU and RSC showed similar trends with a significant reduction in *S. mutans* OD450 nm for 0 and 4 h compared to the control bacteria. This study therefore illustrates that when the GICs are exposed to the *S. mutans* for 2, 4, and 6 h, there was a decrease in the *S. mutans* OD450 nm in relation to the control bacteria. The immediate short term effect of the nanodiamonds are attributed to the carboxylic groups that are among the most common and abundant functional groups [29]. The carboxylic groups present on the nanodiamond surface and participate in ion exchange during the GICs acid–base reactions. The ion exchange occur specifically with Al^3+^ and Sr^2+^ to form various metal salts which can be released upon hydrolysis [30] and provide antibacterial properties to the GICs.

The colonization of plaque in the oral cavity and on various surfaces is complicated and occur in phases. Firstly, the various components of saliva, crevicular fluid, and colonizing bacteria play a role in pellicle formation [31, 32]. The bacterial adherence to the inhabiting environment is essential to their survival in the oral cavity [33]. Studies on the bacterial adherence to GICs and the subsequent biofilm formation on the surface have shown that GICs do influence the bacterial adherence as well as the biofilm formation [13, 34]. The addition of the modifications shown to be effective to reduce the growth in the initial time periods. The biofilm comprises of the initial adsorption of glucan-binding proteins produced from the saliva [31] by *S. mutans* [35] and pioneer bacterial colonies within the pellicle. *S. mutans* synthesize an adhesive glycan from sucrose [36] and in combination with the van der Waals electrostatic forces, create a reversible adherence to teeth. As the various microbes of especially *S. sangius* and *S. mutans* anchor to the pellicle, the plaque becomes visible after 12–24 h. The extra-cellular polysaccharides (EPSs), glucosyltransferases (GTFs) [37] and the various proteins produced by bacteria [38] can all be vulnerable to the influence of the nanodiamond particles incorporated into the GICs. FN has been extensively used for *in vitro* studies on various aspects of research on its properties and clinical success [39] and is therefore an ideal material to have as a control material. The success of the FN material modified with nanodiamonds to reduce the bacterial growth without significant effects on the Ra illustrate the versatility of the material to be modified with nanodiamonds.

**Figure 2:**
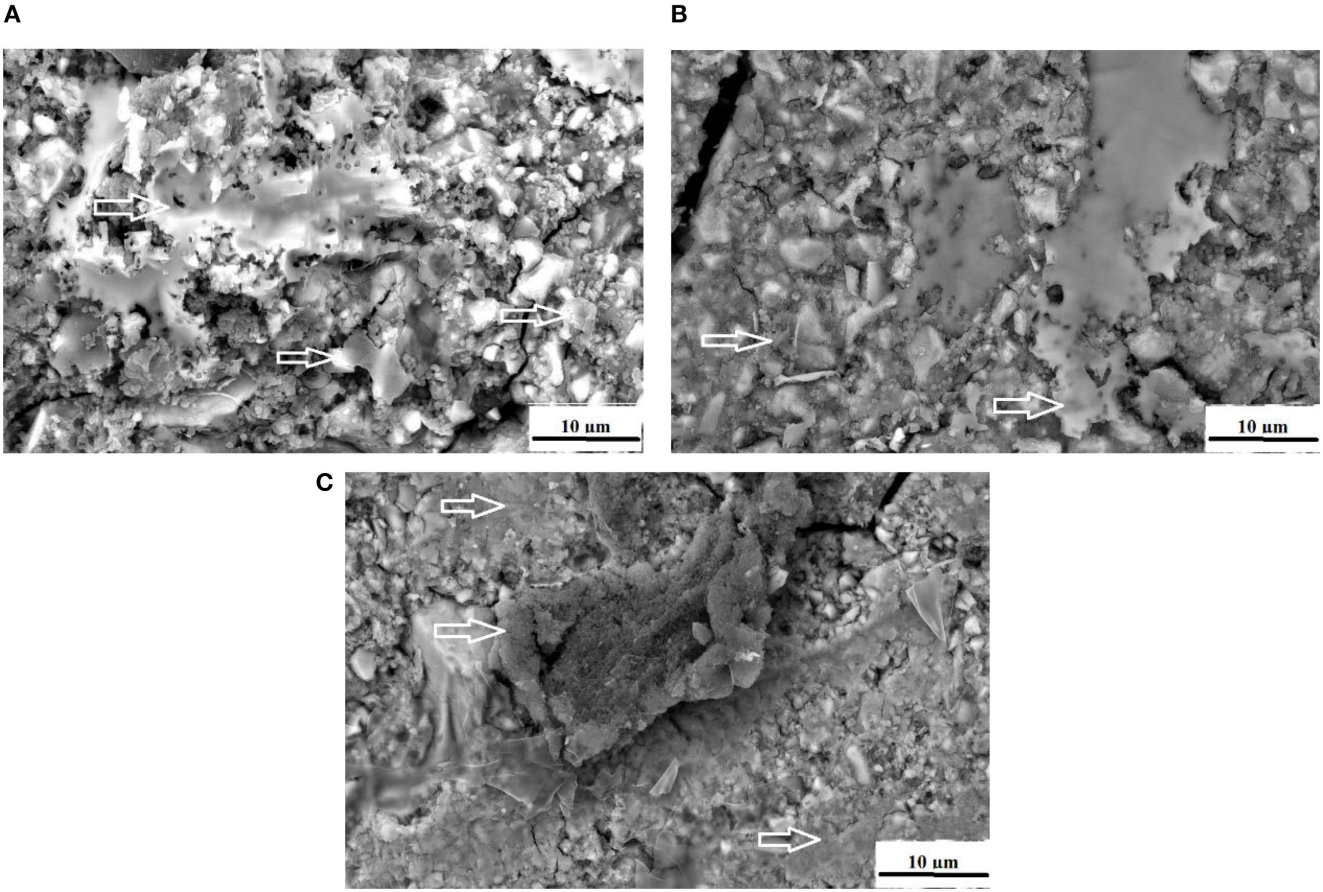
The white arrows indicate the *S. mutans* biofilm on the surface of the control GICs. **(A)** FN present with the thinnest biofilm; **(B)** KU present with thicker biofilm than FN; **(C)** RSC present with a biofilm ranging from transparent to very thick.

At 0 and 24 h, the *S. mutans* OD450 nm were variable between the different chitosan and nanodiamond modifications in relation to the control. With significant differences (*p* < 0.0001) noted between the three control materials at 24 h, it became clear from Figure 2 that the *S. mutans* OD450 nm of FN (0.783) (Figure 2A), KU (0.927) (Figure 2B) and RSC (1.056) (Figure 2C) differed. The arrows indicate *S. mutans* formed biofilms with various thicknesses. The *S. mutans* for the material samples of FN and KU were smaller and more individually spread, with the filler particles of the GICs still visible. For RSC, the biofilm ranged from transparent biofilm to very thick biofilm coverage of the filler particles and matrix of the GICs with many quorum forming *S. mutans*. Figure 2 correlates well with the result at 24 h where the biofilm that formed was the thickest and best developed for RSC followed by KU and then FN with the thinnest biofilm.

The antibacterial effect of the nanodiamond particles originate from the reduction in ATP production of gram-negative *Escherichia coli* to <10%. When compared to the control, this was achieved within 15 min with nanodiamond particles in a concentration of 50 and 500 mg/L. Similar results were noted with the gram-positive bacterium of *Bacillus subtilis* [40]. In the present study, a similar trend was observed with the nanodiamond modifications of FN, KU and RSC where many of the GICs modifications had a significant reduction at 0 h (after 20 min of exposure to *S. mutans*) compared to the *S. mutans* control. Wehling et al. [40], further identified that nanodiamond particles with *E. coli* at a non-lethal dose (0.5 mg/L) were phagocytosed by the bacterium. Wehling et al. [40], concluded that the antibacterial property of these nanodiamonds (PL-D-G01) was not only due to the surface functional groups of the nanodiamonds, but also to the change in the Zeta potential. The loss of the surface reactive functional groups resulted in the reduction of the negative Zeta potential to become more positive. The acid anhydrate functional group was established to be the distinguishing antibacterial group of the nanodiamond particle [40, 41]. In the nanodiamond-modified GICs used in this *in vitro* study, non-covalent interactions occurred between the nanodiamond particles, the ions of the GIC and the polyacrylic liquid [42]. This type of non-covalent interaction would therefore include ionic interactions, hydrogen bonding and dipole–dipole bonding to the nanodiamond particles' (PL-D-G01) terminated carboxyl groups and the other oxygen-containing groups [40, 43].

The nanodiamond particles used in this *in vitro* study were supplied by the manufacturer as non-clustered. Unfortunately it was noted that, the clustering (or rather agglomeration) of the nanodiamond particles did however occur while they were stored in their packaging due to various cohesive and adhesive forces between the nanodiamond particles [44]. This is an unavoidable property of nanodiamonds. These clusters form due to capillary forces pulling the individual nanoparticles together during the drying of the nanodiamonds (for storage) at the time of manufacture [43]. This has physical implications for the incorporation of nanodiamond particles into GICs powder. The advantage of the GICs hand-mixing process was that the agglomerated nanodiamond particles seemed to become smaller during mixing. Upon visual inspection the material seem homogeneously mixed with seemingly small clusters of agglomerated nanodiamond particles noted. The spatulation process separated many of them into micro-sized agglomerated clusters, but the nanoparticle size was not easily achieved the higher the percentage of nanodiamonds became.

**Figure 3:**
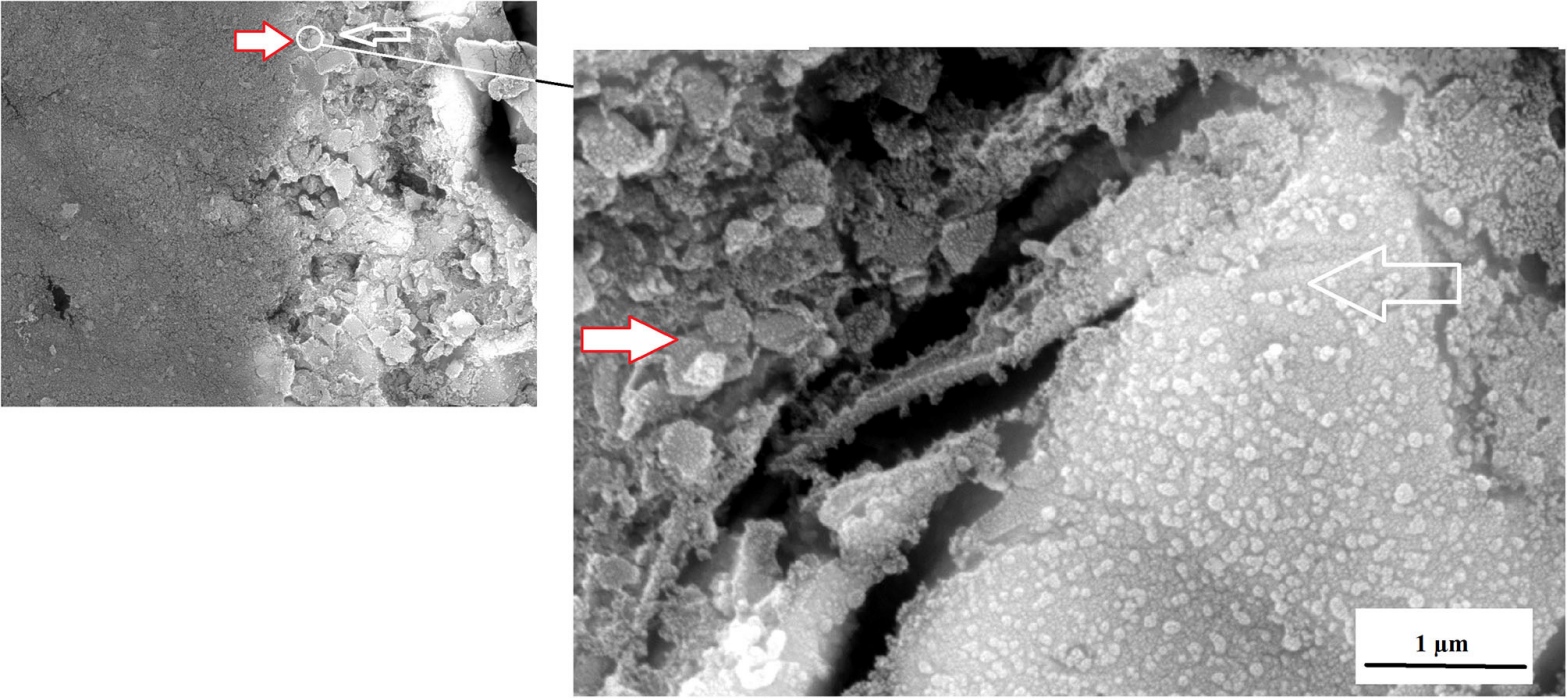
KU5%ND nanodiamond agglomerate (red/white arrow) and the GIC matrix (white arrow).

An advantage of nanodiamonds is the large surface area due to the nano size of the particles. When the nanoparticles agglomerate during the drying process to form a larger “microdiamond agglomerated particle,” there are fewer functional groups around the circumference of the particle that can interact with the ions from the GICs and the forming matrix. The inner particle in the cluster remain shielded from interaction during the acid base reaction. The interaction of these agglomerated nanodiamond particles with the substrate (e.g., GICs), might not be suitable if the powder and liquid do not integrate completely during the mixing process. This integration of the liquid with the nanodiamonds occurs to a point, but the agglomerated nanodiamond particle interaction with the GICs is based on the wetting ability of the liquid to the nanodiamond particles and the rate of polyacrylic acid absorption during mixing. This resulted in the need to assess the surface roughness (Ra) after *S. mutans* exposure. The interaction of the *S. mutans* with the modified GICs material surface was essential to assess the interaction of the incorporated particle for long term success of the modified GICs. Figure 3 illustrates the interaction at the edge of an agglomerated nanodiamond particle (gray surface on the left) to the GIC matrix (right) with the red/white arrow indicating the nanodiamond. In Figure 3, it can be clearly seen that the edge of the agglomerated nanodiamond particle was bound to the GIC matrix. The *S. mutans* at the white arrow is clearly visible on the GIC filler particle with only a few individual *S. mutans* seen on the nanodiamond agglomerated particle, indicated by the red/white arrow (Figure 3).

**Figure 4:**
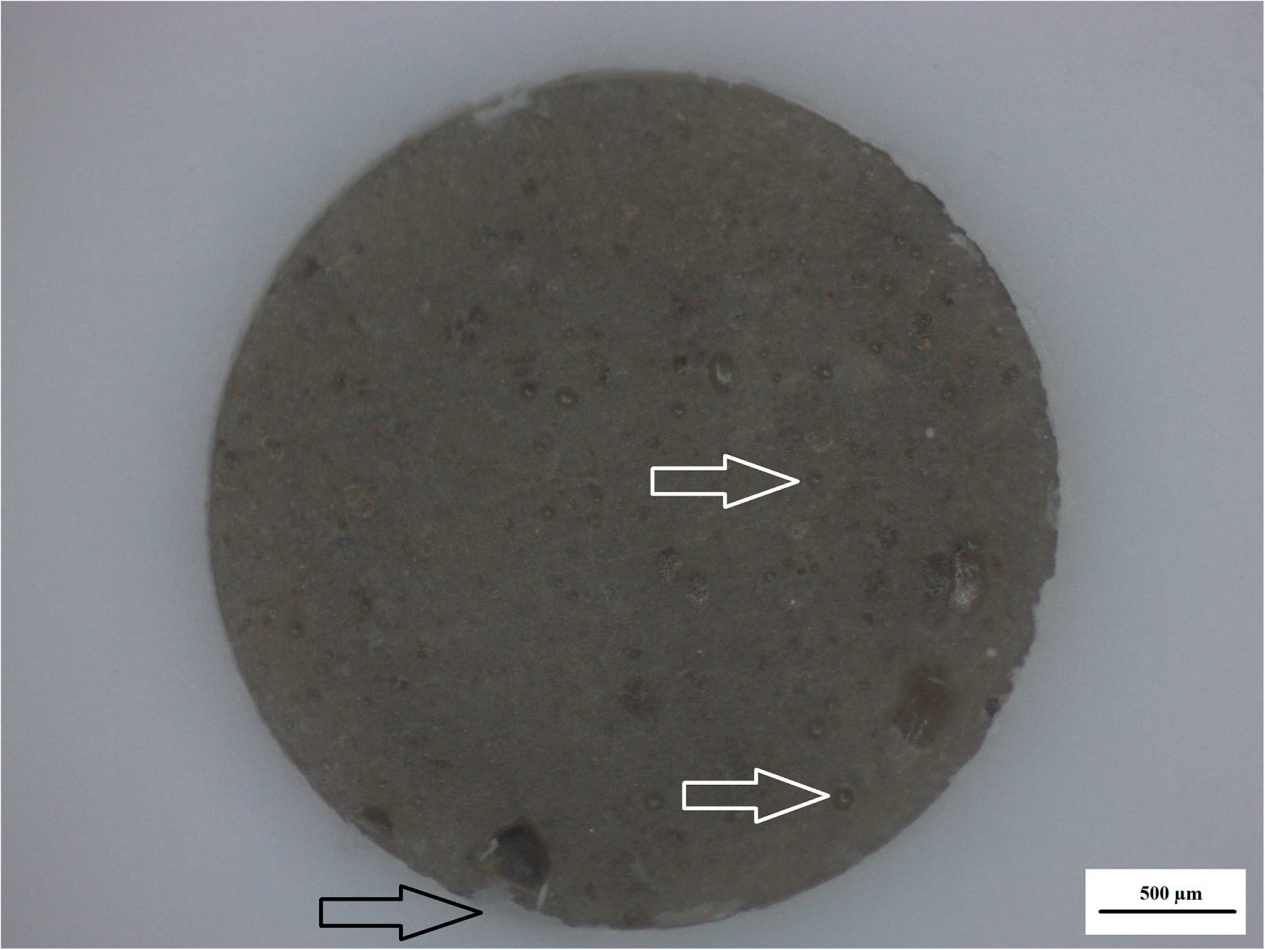
Nanodiamond modified GIC. Nanodiamond cluster lost (black arrow) and nanodiamond particles lost from center of cluster (white arrows).

Surface irregularities like cracks and pits on teeth are the ideal area for early microbial adherence and subsequent plaque formation on the enamel [45]. It has been well documented that the increase in surface roughness of the material result in a deterioration of the material surface [46, 47]. The integrity of the nanodiamond agglomerated particle is dependent upon: (1) the size of the agglomerated nanodiamond particles, (2) the volume of absorbed polyacrylic acid into the agglomerated nanodiamond particle, (3) interaction with the GIC matrix, and (4) the strength of the forces keeping these agglomerated nanodiamond particles together (that were not hydrated with polyacrylic acid). Based on these aforementioned factors the development of voids of various sizes in the center of the nanodiamond-agglomerated particles will occur as the center [29] nanodiamond particles are lost in Figure 4, exposing the GIC surface (indicated by the black arrow). When the nanodiamond particles are lost from the center of the agglomerated nanodiamond particle (indicated by the white arrow), since they are loosely bound with van der Waals forces if the liquid from the GIC does not penetrate into the agglomerated particle. The loss of the nanodiamond-agglomerated particles from the surface of the GIC (indicated by the black arrow) additionally confirmed that the outer margin of the smaller nanodiamond-agglomerated particles (that were moistened by the matrix and the polyacrylic acid) are well-fused with the matrix of the GIC (Figure 4).

**Figure 5:**
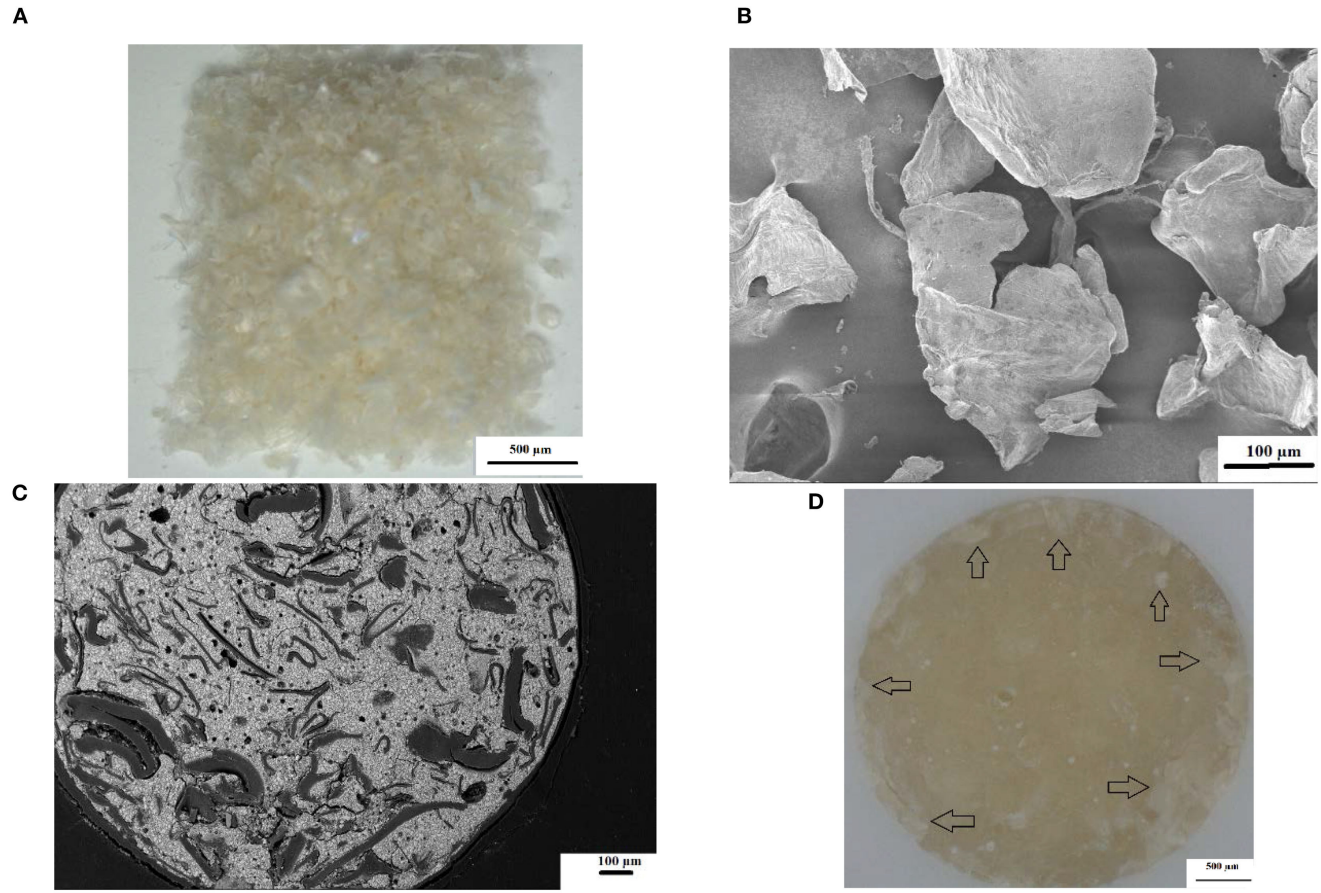
**(A)** Commercial chitosan particles at 6× magnification; **(B)** SEM illustrating the irregular shape of commercial chitosan particles; **(C)** SEM image of KU10%CH illustrating irregular chitosan particle shape and non-fusion with GIC; **(D)** chitosan particles clearly visible as whitish flakes (black arrows).

The surface roughness (Ra) from 0 to 24 h presented with the lowest surface roughness for the 5%ND modification of FN, KU and RSC compared to their respective 10%ND modifications and all the chitosan modifications (Table 1). Although the chitosan modifications had significant increases in the surface roughness, it was attributed to the swelling effect and the interaction of *S. mutans* with the chitosan particles. Therefore, the approach where the chitosan is incorporated into the liquid form remain the most optimal modification for GICs [21–23]. The 24-h exposure of the *S. mutans* to the control materials and their respective nanodiamond modifications did not significantly change their surface roughness (Figure 1). The original particle size of the chitosan material as received from the manufacturer clearly influenced the surface roughness, due to: (1) the larger chitosan particle (Figures 5A,B) which already increase the surface roughness at 0 h compared to the nanodiamond-modified GICs with the smaller agglomerated particles (Figure 1). (2) Their irregular shape (Figures 5B,C). (3) The difference in the chitosan particle distribution [48] within the GICs material (Figures 5C,D). (4) The increase in volume noted of the chitosan as it is exposed to moisture. (5) Non-fusion of the chitosan particle with the GIC (Figure 5D).

**Figure 6:**
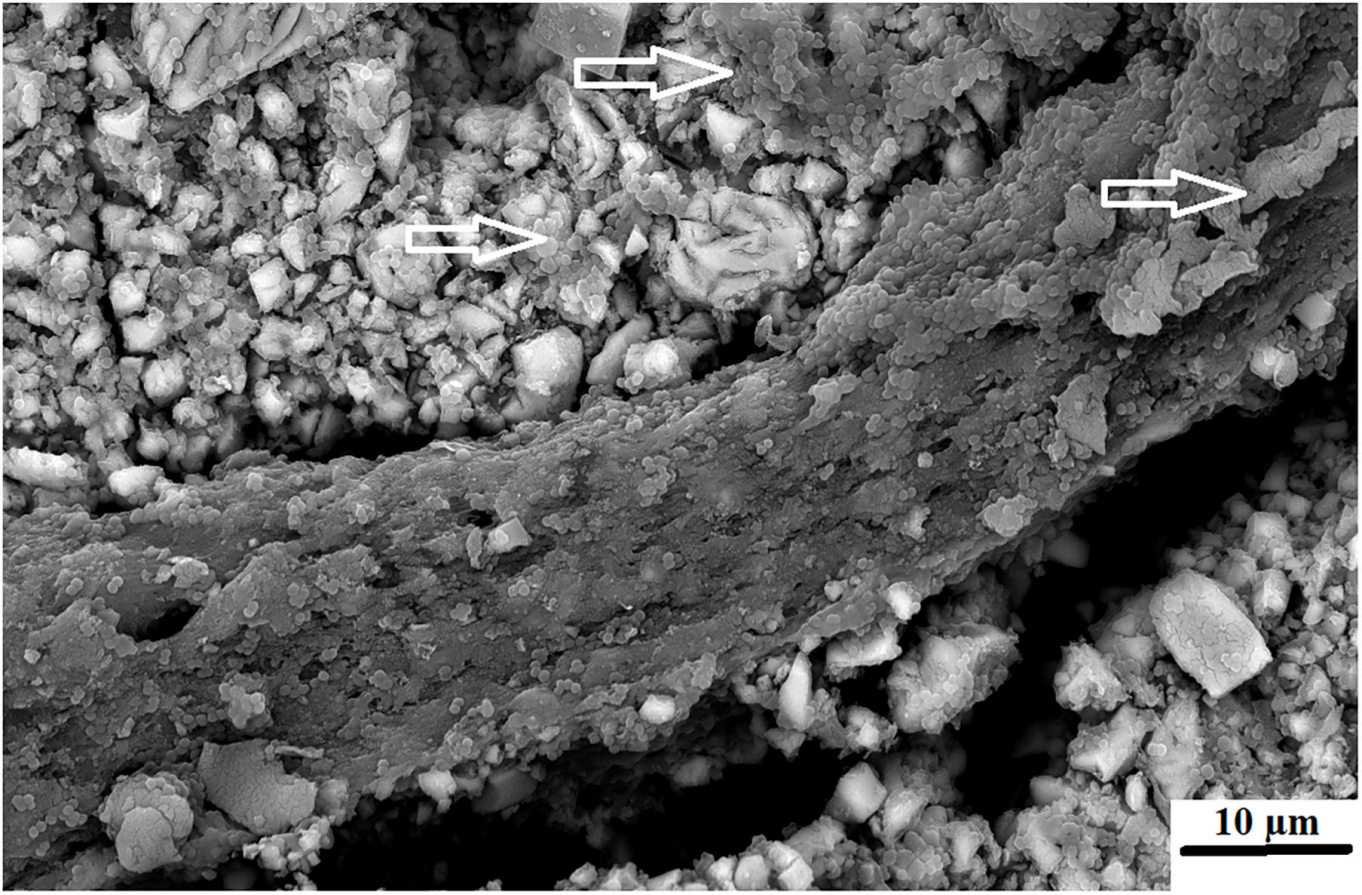
FN5%CH where the chitosan swelled above GIC surface with *S. mutans* visible (white arrows).

The antibacterial properties of chitosan was first noted and applied as a wound dressing for skin and other trauma in 2005 according to the Food and Drug Administration (FDA) registration (Hemcon bandage, Portland, Oregan, USA). Colonization of the chitosan with *S. mutans* is expected to some extent considering that an *in vivo* study has shown that *S. mutans* colonization for various restoration margins is always present, even in different patients [1]. The surfaces of crowns, amalgams and composites have shown consistent *S. mutans* colonization, although the amount of colonization had great variability when the OD450 nm between restorations and the OD450 nm of saliva from the same patients were compared [49]. The chitosan materials seem to have fewer singular *S. mutans* as illustrated by the white arrows highlighting the quorum function in the thick biofilm growth from the chitosan particle (Figure 6).

**Figure 7:**
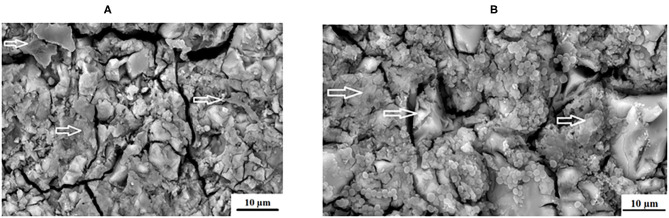
Varied appearance between **(A)**, FN5%ND and **(B)**, FN10%CH with the white arrows indicating the *S. mutans* biofilm.

The individual, quorum forming and various appearances of the biofilm produced by the *S. mutans* in this study appear different on each GICs surface. This could be due to the fact that chitosan was derived from aminopolysaccharide copolymers of glucosamine and N-acetylglucosamine. The chitosan particles contain primary amino groups in the main backbone of the molecule, resulting in the positive charge of the surface [50]. *S. mutans* has a negative charge and is therefore attracted to the surface of the chitosan particle. Further to the amino acids (acetamino acetamide (CH_3_CONH_2_) [51], there are hydroxyl groups at the C2 positions of the glucose ring as well as d-glucosamine that are able to biodegrade and interact with the biological surfaces [50]. Chitosan particles contain many active hydroxyl groups and amino acids that have been attributed to its ability to scavenge hydroxide radicals [52]. This strong scavenging ability of the hydroxyl radicals is important, since it is known to damage amino acids, DNA and proteins [53]. Upon contact with the positively charged chitosan and the negatively charged *S. mutans*, the bacterial cell wall and the protonated ammonia (NH^3+^) from chitosan will compete for the calcium (Ca^2+^). The calcium usually stabilizes the bacterial cell wall and with its reduction, two interferences can occur as cited by a review article by Goy et al. [54]. These include: (1) bacterial cell wall membrane permeability change causing an inhibition of *S. mutans* growth and (2) hydrolysis of the cell wall leading to the leakage of potassium, proteins, nucleic acids, glucose, and lactate dehydrogenase from *S. mutans* [54]. This explains the varied appearance between FN5%ND (Figure 7A) and FN10%CH (Figure 7B).

**Figure 8:**
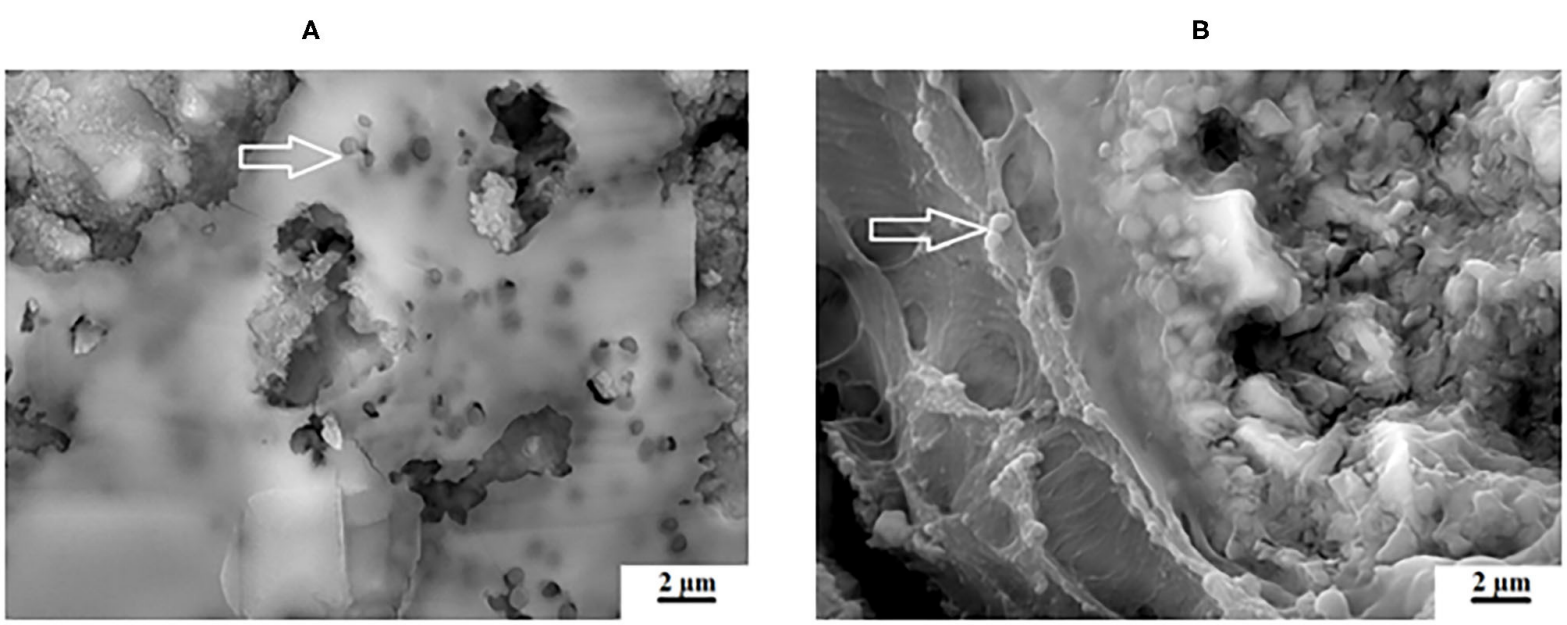
Varied appearance between **(A)**, KU and **(B)**, KU10%CH with the white arrows indicating the *S. mutans* biofilm.

**Figure 9:**
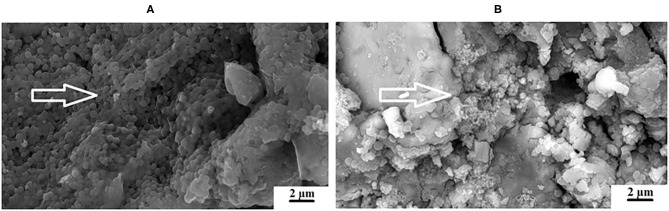
Varied appearance between **(A)**, RSC and **(B)**, RSC5%ND with the white arrows indicating the *S. mutans* biofilm.

The chitosan particles used in the present *in vitro* study was the 75–85% deacetylated particles. In studies [55, 56], the chitosan particles showed anti-bacterial properties, but were dissolved in acetic acid whereas in the present *in vitro* study the chitosan particles were used in the supplied particle form. The only protonation that can occur on chitosan is therefore during the mixing stage as well as the acid–base reaction with the polyacrylic liquid. Even with the ion release from GICs, the adhesion of the *S. mutans* did not seem to be influenced when compared to the other materials [57]. This led the research group of Eick et al. [57], to question the effectiveness of GICs against *S. mutans*. It was well-accepted that fluoride release could influence the *S. mutans* [58], but Montanaro et al. [59], rejected this due to the lack of change in the adhesion of *S. mutans* in relation to other materials tested. To this effect, the adherence of *S. mutans* to the various aspects of the KU (Figure 8A) and RSC (Figure 9A) are illustrated where the biofilm cover the GICs matrix as well as the filler particles. The KU5%CH material modified with chitosan (Figure 8B) and the nanodiamond-agglomerated particles modified into the GIC to form the RSC5%ND material also showed *S. mutans* adherence (Figure 9B).

**Figure 10:**
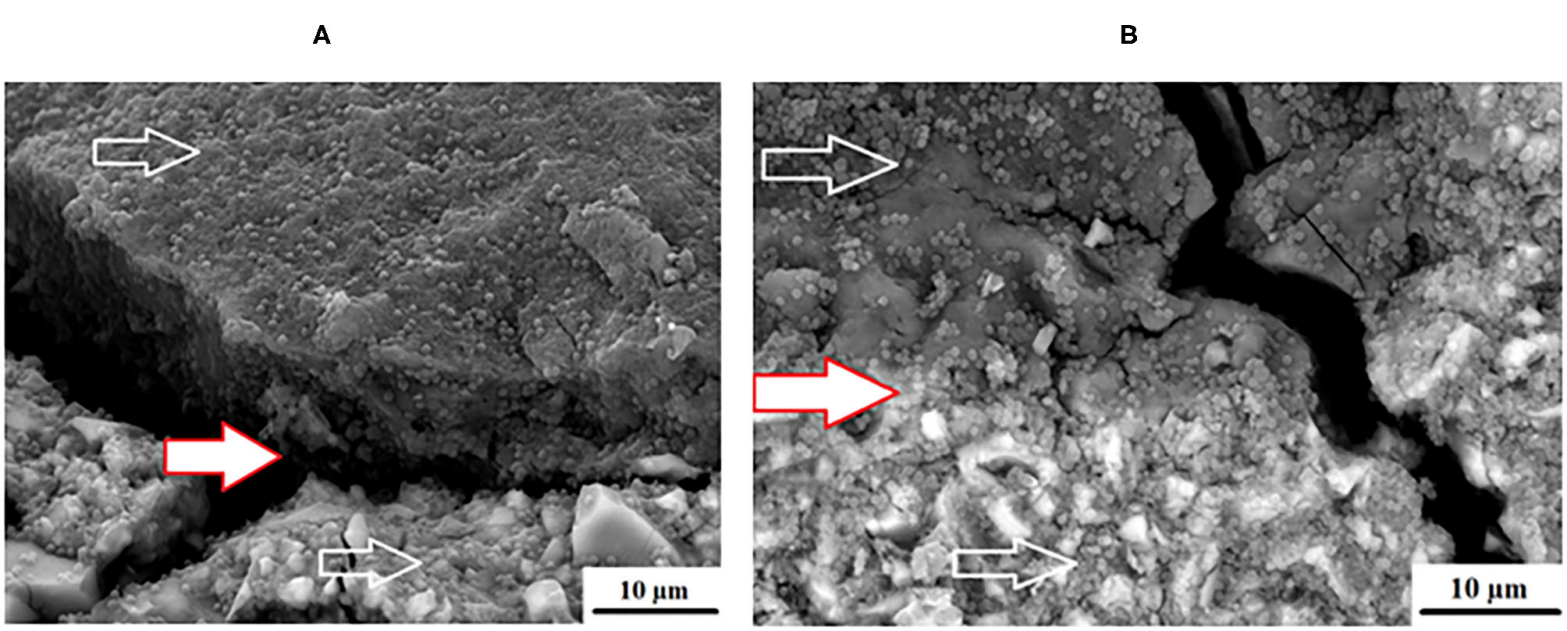
Varied appearance between **(A)**, FN10%ND and **(B)**, KU10%ND with the white arrows indicating the *S. mutans*.

The adhesion level of *S. mutans* to the FN glass ionomer was higher than other regularly used non-GIC dental materials [60]. The presence of *S. mutans* on the GIC surfaces of FN (Figure 2A), KU (Figure 2B) and RSC (Figure 2C) are indicated by the white arrows and the difference in biofilm is visible. The red/white arrows at the nanodiamond/GIC interface indicate that for FN10%ND (Figures 10A,B), the nanodiamond agglomerate separated from the GIC matrix. This did not occur during the sample preparation for the SEM investigation since the *S. mutans* can be seen to colonize well within the space between the nanodiamond-agglomerated particle and the GIC surface. *S. mutans* had therefore been noted to have adherence capabilities and this study confirmed that even in the absence of an initial pioneer pellicle, *S. mutans* as a mono-species was also able to adhere to all the GICs.

With regard to FN, differences were seen between the surface growth of the biofilm and the *S. mutans* at 24 h although there was no significant difference in the *S. mutans* OD450 nm at 24 h between the nanodiamond- and chitosan modifications with their control materials for FN (0.783) (Figure 2A), FN5%ND (0.759) (Figure 7A), and FN10%CH (0.831).

The antibacterial effect for the nanodiamonds are derived from the interaction of the amino acids and the proteins from the growth medium with the nanodiamond particles that reduce the toxic effects of the nanodiamond particles. It has been shown that the nanodiamond particles used in this *in vitro* study (PL-D-G01) exhibit a variety of reactive oxygen groups like acid anhydrides. The acid anhydrides have two acyl groups joined together by an oxygen atom. The other oxygen-containing functional groups such as hydroxyl, alkyl-(derived from sp3-carbon), carboxylic-(C=O) and aromatic groups (C=C) [40, 43] have been noted by other researchers. The various oxygen-containing functional groups add to the antibacterial activities of the nanodiamond particles. The major surface groups of the nanodiamond particles used in this dissertation (PL-D-G01) are therefore carboxylic acid (COOH^−^) and hydroxide (OH^−^) [61]. It has been documented that biological fluids rapidly cover nanoparticles [62]. These terminated carboxyl groups and other oxygen-containing groups of the nanodiamond particles readily interact with amino groups of proteins [42]. Although the antibacterial effect could potentially be overshadowed by the interaction of the growth medium, the *in vivo* situation for nanodiamond particles would be similar since its carboxylic acid groups in the acid anhydride form are highly reactive toward nucleophilic additions [63] with the biofilm and cell membrane of all bacteria.

During the increased *S. mutans* growth over the incubation period, the lactic acid produced by the *S. mutans* interact with the chitosan (Figure 6) that can be readily seen on the surface of the chitosan-modified GICs demonstrating how the chitosan particle has changed when compared to Figures 5A,B. This interaction and the subsequent formation of polyelectrolyte complexes by the chitosan particles [64] provide the required physicochemical properties needed to increase the ion release from the modified GICs causing the chitosan-modified GICs to reduce the *S. mutans* OD450 nm.

The significant reduction of the *S. mutans* OD450 nm at 2 and 24 h in relation to the control *S. mutans* was in line with a study completed with dissolved chitosan and *S. mutans* biofilm growth that was continuously assessed over a 24-h period [65].

It has been noted from the research completed [40], indicated that there could be potential interaction with the growth medium for the *S. mutans* that could inhibit the effects of the chitosan and the nanodiamond particles. Nanodiamond particles have shown to be non-toxic to eukaryotic cells [66]. The non-toxic effect of the nanodiamond particles is due to the affinity of nanodiamond particles to the essential amino acids and proteins. If the nanodiamond particles interact with the essential amino acids and proteins, it would not be able to have a toxic effect on the eukaryotic cells.

This binding of the nanodiamond particles would subsequently inhibit vital enzyme and protein production, decreasing metabolism and resulting in eventual cell death of the bacteria [40]. Due to the negative Zeta potential of the nanodiamond particles, there is usually high dispersion stability in basic aqueous pH solutions. This feature further indicate the presence of carboxylic groups on the surface of the nanodiamond particles [67]. With the surface chemistry of the nanodiamond particles creating a high affinity for amino acid protein adsorption [68]. The carboxylate groups promote the electrostatic breaking of hydrogen bonds between the water molecules. The interaction of water molecules with the carboxylate group results in fewer water molecules in the double hydrogen bond donor configuration and more in a single hydrogen bond configuration [69]. Increased water sorption and the nanodiamond particle becoming more positive as the water sorption occur, result in an increased antibacterial activity as time passes. This matches well with the 24-h reduction of the *S. mutans* OD450 nm compared to the control materials.

The antibacterial effect of chitosan is attributed to its chemical structure, with the presence of deacetylated C2 amino groups which become protonated and positively charged at pH <6.5. Thus, chitosan binds to the membrane of bacterial cells leading to: (1) an increase in membrane permeability with a concomitant increase in the outward flow of ions and proteins from the microbial cell (2) the inhibition of mRNA transcription, and (3) the alteration of protein translation [70, 71]. The molecular weight of chitosan appears to be strongly related to the antibacterial activity of chitosan [72, 73]. The molecular weight may be correlated with the mechanism of destabilization of the bacterial membrane. The lipoteichoic acid that is present in gram-positive bacteria acts as a binding site for chitosan, causing alteration of membrane functionality [74]. In gram-negative bacteria, the chitosan polycations compete with divalent cations and interact with the bacterial cell membrane through electrostatic interactions [75].

The nanodiamond particles provided a greater disruption of the *S. mutans* as illustrated in Figure 9B even though the *S. mutans* OD450 nm was not as low as the chitosan modifications for each material. Nanodiamond particles with the positive influence clearly visible on the nanodiamond-modified GICs surface had reduced surface biofilm compared to the RSC. The biofilm on the nanodiamond-modified GIC is disrupted and not multi-layered. There are also more single *S. mutans* present on the nanodiamond-modified GIC as opposed to a thick multi-cell quorum of *S. mutans* bacteria.

With the biofilm clearly disrupted in the nanodiamond-modified materials, the *S. mutans* would be more loosely bound. This would therefore provide an opportunity for the removal of the loosely bound *S. mutans* during the daily oral hygiene routines of patients.

## Conclusions

The presence of the bacterial biofilm on the margins of restorations had been established as a promoter of secondary caries. Based on the SEM images it became clear that the chitosan and nanodiamond modifications disrupted the appearance of the biofilm and the resultant *S. mutans* growth at 24 h. The control materials and their chitosan/nanodiamond modifications showed significant growth at 6 h compare to the preceding time periods of 2 and 4 h. The materials FN, FN modified with 5% Nanodiamonds, FN modified with 10% Chitosan and KU modified with 10% Chitosan performed the best with regard to the bacterial reduction. Only the chitosan modifications showed an increase in the surface roughness after 24 h of exposure to the *S. mutans*. The chitosan and the nanodiamond modifications provided the best disruption of the *S. mutans* biofilm formation.

### Limitations and Future Recommendations

There was no positive control (e.g., composite, compomer, giomer, or ceramic), nor a negative control (enamel or dentine). The effect of the *S. mutans* and the formation of the biofilm would have added value to the growth and biofilm formation of the *S. mutans* on these substrates to elaborate how it differs from GICs and the modifications. Additional species (e.g., *Streptococcus Salivarious, Lactobacilli, Actinomyces*) could be added in future studies to provide a more complex biofilm on the materials. This more complex biofilm will alter the bacterial film and assess the release of lactic acid for the change in surface roughness of the materials with linear regression analysis. The adhesion of the biofilms could be investigated to provide insight to the direct contact to the restorative materials.

Further research with nanodiamond particles should focus on the incorporation of the nanodiamond particle is in the liquid form to assess if they achieve a greater wetting by the liquid of the GICs. Further this could possibly reduce or eliminate the agglomerated particles completely.

## Data Availability Statement

The raw data supporting the conclusions of this article will be made available by the authors, without undue reservation.

## Author Contributions

RM and EM: conceptualization and methodology. RM: investigation and data curation. EM: resources, supervision, and project administration. RA and RM: writing—original draft preparation and writing—review and editing. All authors have read and agreed to the published version of the manuscript.

## Conflict of Interest

The authors declare that the research was conducted in the absence of any commercial or financial relationships that could be construed as a potential conflict of interest.
